# Effects of a vocational rehabilitation programme on return to work among sick-listed primary health care patients: a population-based matched, case-control study

**DOI:** 10.1186/s12875-020-01123-y

**Published:** 2020-03-27

**Authors:** Anna-Sophia von Celsing, Per Kristiansson, Kurt Svärdsudd, Thorne Wallman

**Affiliations:** 1 Department of Public Health and Caring Sciences, Family Medicine Section, Uppsala University, Uppsala, Sweden; 2 Centre for Clinical Research Sörmland, Uppsala University, Eskilstuna, Sweden

**Keywords:** Multidisciplinary medical assessment, Vocational rehabilitation, Return to work, Sick leave conclusion, Propensity score

## Abstract

**Background:**

To evaluate the efficacy of a multidisciplinary vocational programme in sick-listed, primary health care patients as compared to matched non-programme patients.

**Methods:**

The design was a 3-year prospective population-based, matched case-control study. It was set in a large primary healthcare centre in the city of Eskilstuna, Sweden. The subjects were 943 sickness-certified patients (482 women and 461 men). 170 high-risk patients and a matched control group (*n* = 340) with similar risk for not returning to work within expected time, based on propensity score was created. The intervention group passed a multidisciplinary medical assessment and a coordinated vocational programme, while the control group received usual care by their general practitioner. Main outcome was sick leave conclusion and the day when it occurred.

**Results:**

The follow-up time was subdivided into four periods. During the first two periods, days 1–14 and days 15–112 after baseline, the intervention group had a significantly lower sick leave conclusion rate than the control group (hazard ratios, (HR) 0.32, 95% CI 0.20–0.51, *p* <  0.0001 and 0.47, 95% CI 0.35–0.64). During the third period, days 113–365, the intervention group had an insignificantly lower conclusion rate (HR 0.70, 95% CI 0.46–1.08, *p* = 0.10), and during the fourth follow-up period, days 366–1096, the intervention group had an insignificantly higher conclusion rate than the control group (HR 1.16, 95% CI 0.69–1.96, *p* = 0.58). Across the total follow-up period, the intervention group had a lower conclusion rate than the control group (HR 0.55, 95% CI 0.45–0.66, *p* <  0.0001).

**Conclusions:**

No positive significant effects of the rehabilitation programme on time to sick leave conclusion were found.

## Background

Most sick-certified patients return to work within a short time period, almost 50% are back to work within 14 days [1]. However, long-term sickness absence is a major public health and economic problem in many western countries [2]. A history of prolonged or recurrent sickness absence makes it less likely that the individual will return to work [3–5]. Consequently, early return-to-work programmes are emphasised [6].

In the literature the term ‘return to work’ is usually used to indicate that the sick leave period has ended. However, since not all subjects go back to work when the sick leave period is over, the term ‘sick leave conclusion’ is used in this study in parallel to return to work to indicate that the sick leave period has ended, whether the subject is back to work, or is retired, or is out of a job.

Risk factors for not concluding sick leave within expected time have been evaluated in many studies [7–12]. In an earlier report from the present study, the most important risk factors were age, sick leave diagnosis and sick leave track record during the past year [1]. Having access to the individual risk factor pattern of subjects on sick leave provides the possibility of early identification of patients who may not conclude their sick leave period as expected. In another previous report from this study, a further development of the risk factor concept was presented in the form of nomograms, where the risk of not concluding sick leave within the expected time might be obtained based on the three most important risk factors [13].

Several European countries have varieties of sick leave conclusion programmes that aim at facilitating and hastening sick leave conclusion [14]. These programmes usually include medical management, physical rehabilitation, worker-job matching and managed care. Several studies with specific treatment interventions directed towards special target groups, usually with musculoskeletal problems, have shown positive effects on return to work [14, 15]. However, so far, no multidisciplinary rehabilitation intervention directed towards patients with any cause of sick leave, has been shown effective regarding sick leave conclusion in the general population [16].

A problem with previous multidisciplinary rehabilitation interventions is that the rehabilitation was introduced rather late in the sick leave period, by which important time in a return to work programme was lost. Another problem has been rather small study populations with a resulting low statistical power. For this reason we decided to perform the present multidisciplinary rehabilitation intervention in a large study population with the hypothesis that early assessment of patients at risk of long-term sick leave and early onset of multidisciplinary rehabilitation might reduce days of sick leave and thus hasten return to work.

## Methods

### Setting, design and study population

The National Social Insurance covers all Swedish permanent residents between 16 and 65 years of age, whether citizens or not [17]. The insurance covers access to primary or hospital care at heavily subsidised rates, the right to see any physician of one’s own choosing, to have sickness benefits for income loss in case of reduced work capacity due to injury or disease and many other benefits. At the time of the study, there was no limit of the time a patient could be sick-listed.

The study protocol used in this study has been described previously [1]. Briefly, the study was designed as a three-year prospective, cohort study and was performed at one of the primary health care centres in Eskilstuna, Sweden, with 10 general practitioners serving a population of approximately 25,000 residents. A total of 943 patients (482 women and 461 men), who were 18 to 63 years of age, sickness-certified by a general practitioner at the centre at any time from 1 January until 31 August 2004 and who gave their informed consent of participation were included in the study. Patients already included in a medical or vocational rehabilitation programme were excluded.

### Baseline data

Baseline data obtained from the sickness certificate included the age, sex, occupational status (in gainful work or not), sick leave diagnosis according to the WHO International Classification of Diseases (ICD-10) [18] and degree of sick leave (25, 50, 75% or 100%). Information regarding the sickness absence track record during the 365 days preceding the baseline examination was obtained from the National Social Insurance Agency database. The data included sick leave diagnoses, first and last day of each sick spell, information on marital status, salary, whether born in Sweden, and for immigrants, Swedish citizenship status.

At the time of the study, patients could self-certify the first 7 days of a sick leave. If the sick leave protracted beyond this point, a physician’s sickness certificate was needed. For this reason, the sick leave information was verified with the primary healthcare medical records and completed with self-certified days.

A manual classification of the chances of concluding the ongoing sick leave period on expected time was made based on any of the following variables: a sickness certification track record during the last year of more than 28 days, being sickness-certified at baseline because of musculoskeletal disease (ICD code M) or a psychiatric disease (F), being unemployed, being older than 45 years and being a woman. Of the 943 patients, 496 were classified as low risk of not concluding their sick leave on time, 277 as having a moderately high risk and 170 as having a high risk.

### Intervention

**Fig. 1:**
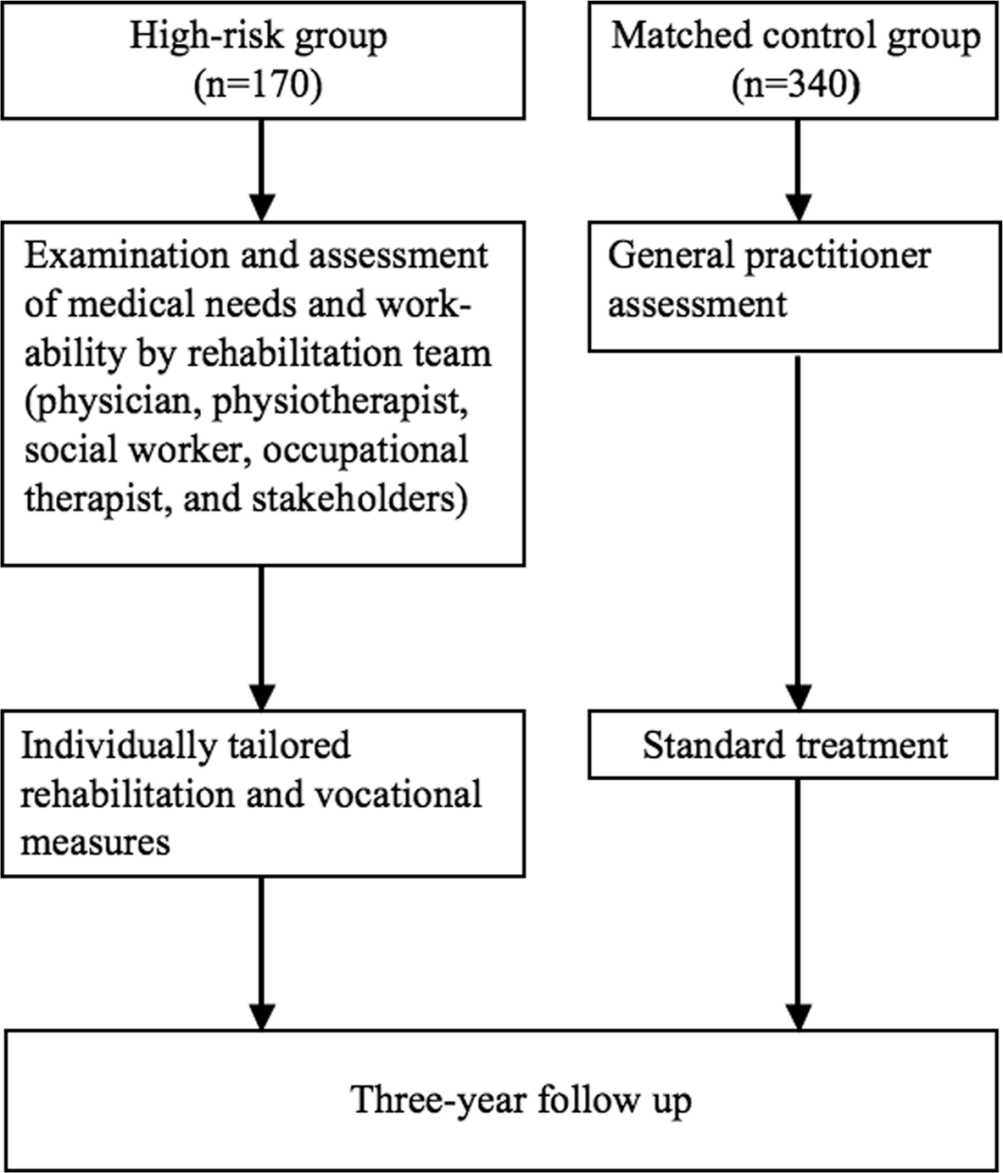
Flow-chart of assessment and intervention

The intervention programme started within 1–3 weeks from baseline with medical examinations by all members of the multidisciplinary team, including a physician, a physiotherapist, an occupational therapist and a social worker (Fig. 1). Medical needs and workability were assessed in a case discussion within the team, after which an individually tailored training programme started. This typically included physical exercise, physiotherapy treatment, occupational therapy, CBT, stress and pain management. There were no group sessions performed. There were no specific workability questionnaires used. The individual assessment of workability was based on the results of the medical examinations and dialogue with the patient.

At the same time, the individually coordinated vocational programme, as agreed with the patient, was implemented by the multidisciplinary medical team and the stakeholders from the local Social Insurance Agency, the local Employment Agency and a social worker from the city of Eskilstuna. For employees this typically included an ergonomic assessment, identification of barriers for return to work, regular appointments with stakeholders and meetings at the workplace. The purpose was to identify problems perceived as hindering return to work and finding solutions, like gradual return to work, modifications of work, workability training or change of work. For non-employees, the programme was based on the individual assessment of workability and help from the local Employment Agency to find a suitable regular work or a modified work. Meetings on a weekly basis with all involved stakeholders ensured that all patients received the same basic intervention, including examination and assessment by the multidisciplinary team and an assessment with the stakeholders. However, the vocational programme was individually tailored based on the specific needs of the patient and the given possibilities within the programme.

The 773 subjects with moderately high or low risk received standard treatment as recommended by their general practitioners.

### Follow-up data

Information on sickness absence during the 3-year follow-up from baseline was obtained from the National Social Insurance Agency database, including sick leave diagnoses and first and last day of each sick spell, and whether a disability pension was granted during follow-up. Information on vital status and date of death for those who died (*n* = 6) was obtained from the National Cause of Death register.

### Statistical considerations

Data was analysed with the Statistical Analysis System (SAS) software, version 9.3. No data were missing. The outcome of the study was sick leave conclusion and the day after baseline when it occurred. Degree of sick leave was not taken into account, since only 10% had less than full sick leave.

In order to decide on the day of sick leave conclusion (or return to work) the method proposed by Bogefeldt et al. was used [19]. The three-year sick-leave follow-up period was converted into a day-by-day matrix starting with variable ‘day 1’ (baseline day) and ending with variable day ‘1096’ (end of follow up). Each variable measured whether the subject was on sick leave (**=** 1) or not (**=** 0) on that day.

Based on this matrix a sick leave conclusion variable for the sick leave period in effect at baseline was computed. Two criteria were applied for each sick spell: (a) the sick spell was followed by a sick-leave-free interval of more than 28 days, regardless of the length of any following sick spell; (b) the sick spell was followed by a sick-leave-free interval of more than 7 days, and that interval had to be longer than the next sick spell. When at least one of the criteria was fulfilled, sick leave conclusion was presumed to have occurred on the first non-sick-leave day. If none of the criteria were satisfied at the end of follow up, sick leave conclusion presumably had not occurred.

The study population was not randomised into an intervention and a control group, initially, since the study was not intended to be a scientific one. A post hoc control group, as similar to the intervention group as possible, was created by means of a propensity score. Propensity score, first proposed by Rosenbaum and Rubin in 1983 [20], implies that matching may be performed based on an unlimited number of matching variables that are weighed together into a propensity score.

A prerequisite for matching in this case was that the manual classification of individuals into a high-risk group versus a moderate-to-low-risk group was imperfect, as it usually is when no explicit variable weights are used. When logistic regression was used to compute predicted risk based on the same variables that were used manually, but now graded (given weights), a substantial overlap of risk score was found, primarily between the high-risk and the moderate-risk groups.

This circumstance then allowed computation of a propensity score with nominal logistic regression using the rehabilitation (high-risk) group (code 1) and all others (code 0) as dependent variable, and age, sex, number of sick leave days last year, sick leave diagnosis, degree of sick leave, whether born in Sweden, whether a Swedish citizen and marital status as independent variables. Based on an analysis of the impact of the various sick leave diagnoses on sick leave conclusion previously published [1], the latter were ranked from **−** 2 (largest impact) to + 3 (least impact). In this way, all variables entered into the logistic regression assumed to carry a risk for not concluding sick leave at the expected time, were collected into one measure, the propensity score.

Subjects in the non-rehabilitation group were then matched to subjects in the rehabilitation group by propensity score to form potential control groups. Mean (standard deviation, SD) propensity score in the rehabilitation group was 0.301 (0.173), in the first matched control group 0.293 (0.162), in the second control group 0.223 (0.100), in the third control group 0.119 (0.034) and in the fourth control group 0.051 (0.034). The scores of the first and second control groups were thus fairly similar to the rehabilitation group and were combined into a common control group. The rehabilitation group (*n* = 170) and the control group (*n* = 340) constituted the study population of this report.

Simple differences between the rehabilitation and the control group were tested with Student’s t-test for continuous variables and the chi-squared test for discrete variables. According to the SAS ‘life test’ procedure, there were no close proportional hazards regarding sick leave conclusion across the total follow-up time. The latter was therefore divided into days 1–14 (when rehabilitation activities had not started), days 15–112 (when most rehabilitation activities were performed), days 113–365 days (when most rehabilitation activities were finished), and days 366–1096 (long-term follow up). For each of these partial follow-up periods, the hazard rates were approximately proportional.

The effect of the vocational rehabilitation programme was evaluated with conditional proportional hazards regression, one analysis for each partial follow-up period, where conclusion of sick leave and the time when it occurred were entered as dependent variables, and group allocation was entered as the independent variable, as well as individual propensity scores to further adjust for the potential remaining risk differences between the groups. To check the results for dependence on remaining propensity score differences, the analyses were repeated using only the first matched group as the control group. The results were the same as shown below, except that measures of dispersion were somewhat wider.

The analysis provided hazards ratios (rehabilitation group versus control group) and 95% confidence limits, Wald’s chi-squared (a measure of exposure impact on outcome) and *p*-values. All tests were two-tailed, and the significance level was set at *p* <  0.05.

## Results

There were no significant differences amongst the baseline demographic variables between the groups (Table 1). However, the intervention group had a larger number of sick leave days during the 365 days preceding baseline and a lower number of sick leave diagnoses with a large effect on sick leave conclusion than the control group. The propensity score, in which all baseline differences were combined, was moderately higher in the intervention group than in the control group.

**Table 1:** Baseline characteristics of the study population

	Study groups				
	Intervention group		Control group		*p*
	n	Mean (SD) or %	n	Mean (SD) or %	
N	170		340		
Age at baseline, years		41.1 (10.4)		40.3 (10.8)	0.41
Male sex, %	74	43.5	148	43.5	1.00
Born in Sweden, %	135	79.4	259	76.2	0.41
Swedish citizen, %	161	94.7	320	94.1	0.79
Marital status, %					0.39
Never married	63	37.1	135	39.7	
Married/Cohabiting	57	33.5	118	34.7	
Divorced	47	27.7	80	23.5	
Widowed	3	1.8	7	2.1	
Sick leave days last year		113.4 (132.2)		69.7 (113.5)	< 0.0005
Sick leave diagnosis^*^					< 0.05
Score < 0, %	148	87.1	301	88.5	
Score ≥ 0, %	22	12.9	39	11.5	
Propensity score	170	0.301 (0.173)	340	0.259 (0.139)	< 0.005

^*^Based on separate analyses the sick leave diagnoses were given weights according to their association with duration of the sick leave period, low weights indicating protracted sick leave period. ICD-10 codes F (psychiatric disorders) and G (neurological disorders) were given the weight **−** 2, codes I (cardiovascular disorders), K (gastrointestinal disorders), and M (musculoskeletal disorders) weight **−** 1, codes A and B (infectious disorders), O (obstetric disorders), and L (dermatological disorders) weight + 1, code N (urogenital disorders) weight + 2, codes H (ophthalmologic or otology disorders) and J (pulmonary disorders) weight + 3, and all other diagnoses codes as 0

The results of the proportional hazards regression are shown in Table 2. During the first two periods, 1–14 days and 15–112 days of follow up, the intervention group had a significantly lower sick leave conclusion rate than the control group (*p* <  0.0001). In the third period, 113–365 days, the intervention group still had a lower sick leave conclusion rate than the control group, even though insignificant (*p* = 0.10). In the fourth follow-up period, 366–1096 days, the intervention group had an insignificantly higher sick leave conclusion rate than the control group (*p* = 0.58). During the total follow-up time the intervention group had a significantly lower sick leave conclusion rate than the control group.

**Table 2:** Effects of rehabilitation versus standard treatment on sick leave conclusion

	Conditional proportional hazards regression analysis						
Follow-up, days	n	Exposure	Parameter estimate (SD)	Wald’s χ^2^	HR^*^	95% CI	*p*
1–14	151	Intervention vs control	−1.15 (0.24)	23.7	0.32	0.20–0.51	< 0.0001
15–112	196	Intervention vs control	−0.75 (0.15)	23.6	0.47	0.35–0.64	< 0.0001
113–365	85	Intervention vs control	−0.35 (0.22)	2.6	0.70	0.46–1.08	0.10
366–1096	78	Intervention vs control	0.15 (0.27)	0.30	1.16	0.69–1.96	0.58
Total follow up	510	Intervention vs control	−0.60 (0.10)	38.3	0.55	0.45–0.66	< 0.0001

^*^Hazards ratio. Adjusted for triplet matching number (conditional analysis) and for remaining propensity score differences

There was thus no evidence that the rehabilitation group would have a significantly faster rate of sick leave conclusion than the control group. When sick leave was concluded the patients might return to work, become unemployed or be granted a disability pension. As shown in Table 3, ‘return to work’ dominated during the first period in both the intervention and the control group and then successively became less prevalent, while ‘unemployment’ and ‘disability pension’ successively increased.

**Table 3:** Occupational status at sick leave conclusion

	Status when present sick leave period ended					
	Return to work		Unemployment		Disability pension	
Follow-up, days	Interv group, n (%)	Control group, n (%)	Interv group, n (%)	Control group, n (%)	Interv group n (%)	Control group, n (%)
1–14	19 (90.5)	105 (80.8)	2 (9.5)	24 (18.5)	0 (0)	1 (0.8)
15–112	42 (67.7)	103 (76.9)	18 (29.0)	29 (21.6)	2 (3.2)	2 (1.5)
113–365	17 (42.5)	27 (60.0)	15 (37.5)	11 (24.4)	8 (20.0)	7 (15.6)
366–1096	12 (25.5)	19 (61.3)^1^	11 (23.4)	3 (9.7)	23 (48.9)	9 (29.0)

Interv = intervention, ^1^*p* < 0.005

## Discussion

The results of this study indicated that the intervention group had a significantly lower sick leave conclusion rate than the control group during the first two follow-up periods, an insignificantly lower rate during the third period and an insignificantly higher rate during the fourth period, after adjustment for risk factor differences between the groups. Furthermore, the proportion of subjects who returned to work was high during the first two follow-up periods and then successively decreased, while unemployment and disability pension successively increased.

It has been suggested that early multidisciplinary medical rehabilitation and coordinated vocational intervention are generally effective and are recommended to be included in all interventions to enhance return to work [21, 22]. Several studies with specific treatment interventions directed towards target groups have shown positive effects [14, 15]. However, several previous studies, with interventions similar to the present one, have evaluated the effects of rehabilitation on sick leave conclusion in sickness-certified patients. We found six randomised controlled trials (RCT) [16, 23–27], one study with a matched two-cohort design [21], and one review [28]. Johansson et al. [16] concluded that the intervention prolonged sickness absence spells, as we did. Haldorsen et al. [23] found a positive effect in subjects with a moderately bad or a bad prognosis but no effect in the good prognosis group. We found no effect of prognosis (measured as propensity score). Carlsson et al. [24] and Jensen et al. [25] found no significant difference between the intervention and control groups. Anema et al. [26] found a positive effect of workplace intervention in subjects with low back pain but no effect of graded activity. The Danish return-to-work programme [27] found that a multidisciplinary intervention did not facilitate return to work or decrease health care utilisation as compared to ordinary case management in patients with somatic symptoms, anxiety or low self-rated health. Suoyrjö et al. [21] found that during 7 years of follow-up, the intervention group had more sick leave days than the controls, as in the present study. Vogel et al. [28] found no benefit for return-to-work programmes on return-to-work outcomes during 12 months of follow-up as compared to usual practice.

In Sweden, multidisciplinary and vocational rehabilitation, in coordination with workplaces and authorities, is the recommended method to promote return to work. However, this recommendation does not appear to be supported by scientific evidence. As pointed out by Johansson et al. [16] and Carlsson et al. [24] the rehabilitation team may focus more on rehabilitation than on encouraging the sick-listed individual to return to work, and the early intervention programme may therefore have a locking-in effect, i.e., actually preventing the sick-listed from concluding sick leave and returning to work. The results from the present study may be due to such an effect.

Another possible explanation might be that it was a prerequisite to be sick-listed by a physician to be entitled to rehabilitation measures taken by the Social Insurance Agency. The coordination of different involved stakeholders was also a time-consuming process. Moreover, all involved stakeholders may have had their own agenda. The patient needed a job or some sort of wage/subsidy for his/her subsistence, the physicians wanted to restore the sickness-certified person’s work capacity and the Social Insurance Agency may have aimed at keeping costs within budget frames. These agendas may not be completely compatible or even compatible at all.

An important factor might be the timing of rehabilitation. In a United Kingdom review, it was found that in the first 3–6 weeks of sick leave, the likelihood of recovery and rapid sick leave conclusion is high, with or without healthcare intervention [29], as was found in another report from the present study [13]. After 6 weeks of sickness absence in workers, the risks of long-term incapacity increased by 10–20%, and after 6 months, there was only a 50% chance of returning to a previous job. Moreover, the sickness-certified subject should be more involved in the rehabilitation process, since the patient’s own prediction of length of sick-leave, motivation and belief has been shown to have a positive impact on return to work [30–32].

The strengths of this study were that the study population covered all patients who were sickness-certified during a certain period (time window), which means that the study population might be regarded as equivalent to a random sample of the local sickness-certified population. Moreover, the exposure and outcome data used in the analyses were obtained from official sources, such as sick leave certificates, medical records and the national Social Insurance Agency database. Data were complete with no losses.

Furthermore, the data on which sick leave conclusion and return to work were based have high face validity. Another strength was the similarity to everyday clinical practice in a primary health care centre, with its diverse patient population and rehabilitation based on existing professions as well as meetings on a regular basis with stakeholders from the authorities.

In 2004, multidisciplinary rehabilitation was state of the art in Sweden in return to work programmes for patients on sick leave. The involvement of the employer and stakeholders from authorities in the vocational rehabilitation was generally recommended. However, in reality this did not happen until the Social Insurance Agency observed that a patient had been on sick leave for about 6–12 weeks. Therefore, in this study, bringing the stakeholders from authorities to the primary health care centre for meetings on a weekly basis tested a new method. The aim was to save time by initiating coordinated measures for return to work as early as possible in a sick leave period. This working method was intended to be pragmatic and closely linked to the daily clinical practice within primary health care. However, the effect of it remains inconclusive and a weakness is the lack of randomisation.

A limitation of the study was that a randomised, controlled trial was not possible to perform, since the study initially was not meant to be a scientific one. Some sort of matching was therefore necessary to obtain a control group as similar to the intervention group as possible. We chose to base the matching procedure on the propensity score method, which provides an individual score for not concluding sick leave as expected, based on well-known and generally accepted risk factors. After matching, there were still moderate differences in the propensity score between the groups. By adjustment in the analyses for these remaining propensity score differences, results similar to the random allocation were obtained.

A further limitation might be that the analyses and results are based on data collected in 2004–2007. However, despite changes in business cycles, and financial initiatives from the Swedish government to improve rehabilitation and sick leave management promoting return to work, the results from recent rehabilitation and vocational intervention studies show results similar to the present one [16, 24, 25, 27, 28].

The results from the present study indicate that treatment-as-usual, as performed by the GPs, might be as efficient on return to work, as a multidisciplinary coordinated vocational programme including involvement of stakeholders and employers. It has also been shown that specific rehabilitation and vocational interventions directed towards target diagnose groups facilitate return to work. However, a standard intervention does not seem to be efficient for all patients in a primary health care context with its diverse patient groups. More research is needed on the content of the GPs’ ‘treatment as usual’ to promote return to work. A qualitative GP interview-study would be of interest. How do GPs think? What interventions do they choose and why? Easily available ones? Referrals and/or personal contacts with stakeholders and employers? Importance of the patients’ own assessment of workability? How does the GP assess workability? It is also important to involve the patients in the return to work programme. A recommendation for a future study would then be to let the patients decide their own rehabilitation and return to work programme in a randomised controlled trial with cases receiving an individualized programme after the patients’ own choice and controls receiving treatment as usual.

## Conclusions

There was no evidence in this study of an effect on sick leave conclusion of an early multidisciplinary medical assessment and coordinated vocational rehabilitation programme among primary health care patients on sick leave. Results from other similar studies primarily followed the same trend. Facilitation of the sick leave conclusion process concept should be reconsidered. It might be argued that implementation of such a complex coordinated vocational programme is unnecessary and of low cost-benefit. From a medical and economic point of view, a simple standard treatment as prescribed by the physician appears to be a better choice. Moreover, the sickness-certified subjects should be more involved in this process, since the patients’ own prediction of length of sick leave, motivation and belief has been shown to have a positive impact on sick leave conclusion.

## Data Availability

The datasets used during the current study with anonymous observations are available from the corresponding author on request.

## Abbreviations

WHO: World Health Organization
ICD: International Classification of Diseases, 10th revision
GP: General Practitioner
SAS: Statistical Analysis System
